# Attention-Guided Network with Densely Connected Convolution for Skin Lesion Segmentation

**DOI:** 10.3390/s21103462

**Published:** 2021-05-16

**Authors:** Shengxin Tao, Yun Jiang, Simin Cao, Chao Wu, Zeqi Ma

**Affiliations:** College of Computer Science and Engineering, Northwest Normal University, Lanzhou 730070, China; jiangyun@nwnu.edu.cn (Y.J.); 2018221813@nwnu.edu.cn (S.C.); 2019211754@nwnu.edu.cn (C.W.); 2019221847@nwnu.edu.cn (Z.M.)

**Keywords:** deep convolutional neural network, skin lesion segmentation, attention mechanism, computer-aided diagnosis

## Abstract

The automatic segmentation of skin lesions is considered to be a key step in the diagnosis and treatment of skin lesions, which is essential to improve the survival rate of patients. However, due to the low contrast, the texture and boundary are difficult to distinguish, which makes the accurate segmentation of skin lesions challenging. To cope with these challenges, this paper proposes an attention-guided network with densely connected convolution for skin lesion segmentation, called CSAG and DCCNet. In the last step of the encoding path, the model uses densely connected convolution to replace the ordinary convolutional layer. A novel attention-oriented filter module called Channel Spatial Fast Attention-guided Filter (CSFAG for short) was designed and embedded in the skip connection of the CSAG and DCCNet. On the ISIC-2017 data set, a large number of ablation experiments have verified the superiority and robustness of the CSFAG module and Densely Connected Convolution. The segmentation performance of CSAG and DCCNet is compared with other latest algorithms, and very competitive results have been achieved in all indicators. The robustness and cross-data set performance of our method was tested on another publicly available data set PH2, further verifying the effectiveness of the model.

## 1. Introduction

One third of cancers worldwide are skin cancers [1]. Currently, 2 to 3 million cases of non-melanoma skin cancer and 132,000 cases of melanoma skin cancer occur globally each year. It is estimated that in the United States, there were 96,480 new cases and 7230 deaths from melanoma in 2019. Melanoma accounts for less than 5% of all skin cancers, but 75% of skin cancer deaths are related to melanoma. Studies have shown that the 5-year survival rate of patients with advanced malignant melanoma is only 15%, while the final cure rate of early patients is as high as 95% [2]. Patients with benign melanoma only need to be found early and removed to prevent the disease from being life-threatening [3]. Therefore, the diagnosis of benign and malignant melanoma as well as early and late stages can play an extremely important role in the survival of melanoma patients.

**Figure 1:**
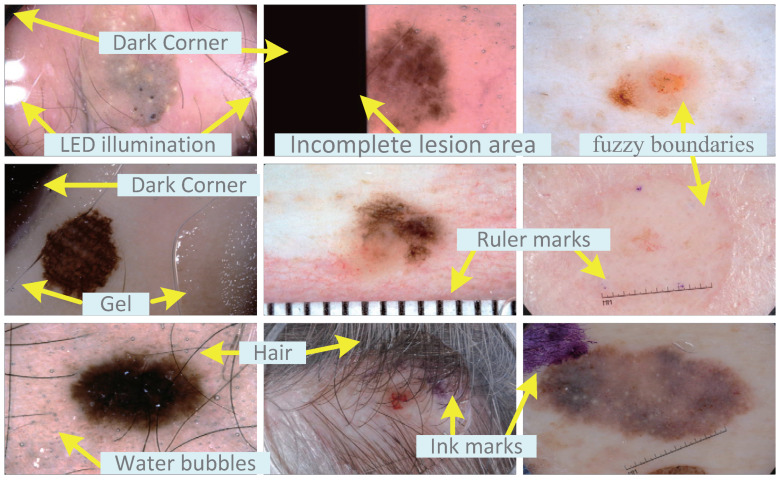
A typical pictorial presentation of the skin lesion images with different challenging for segmentation.

Dermoscopy is an image formed based on the imaging principle of removing the reflection of the skin surface and visually enhancing the deeper skin [4]. Compared with visual inspection, dermoscopy can improve the diagnostic accuracy rate by 20% [5]. Skin lesion segmentation is one of the important steps in the computer-aided diagnosis of various skin diseases. As shown in Figure 1, due to the huge differences in the size, location, shape and color of the lesions in different patients, and a large number of artifacts, including inherent skin features (such as hair, blood vessels) and artificial artifacts (such as bubbles, ruler marks, uneven lighting, incomplete lesion areas, etc.), the automatic segmentation of lesions in dermoscopic images is very challenging. In addition, the low contrast between the lesion and the surrounding texture also hinders the automatic segmentation of the lesion area.

With the help of computer-aided diagnosis, segmentation of skin lesion images to obtain lesion areas helps doctors quickly identify the location of the lesion area, which can improve the diagnosis rate and enable patients to be treated early. In the early stage, the skin lesion segmentation image use edge detection, threshold segmentation, active contour (expectation maximization, level set, clustering, etc.) or region-based (region growth, iterative stochastic region merging, etc.) hybrid technology [6,7]. Although the above segmentation algorithm has a certain segmentation effect, it relies too much on the quality of manual feature selection and introduces prior information. Moreover, it is difficult for the recognition model based on artificial features to obtain good generalization ability for skin lesions images with highly changed clinical manifestations.

Recently, supervised methods have achieved promising results in the field of computer vision, but they rely on annotated training data sets, which require the proficiency of humans and related background knowledge. In contrast, unsupervised learning makes data-driven decisions by obtaining insights directly from the data itself. Unsupervised learning is applied to all aspects of image processing. Ahmed et al. [8] proposed a low-rank tensor with a sparse mixture of Gaussian (LRTSMoG) decomposition algorithm for natural crack detection. He proposed algorithm models jointly the LRST pattern by using a tensor decomposition framework. In particular, the weak natural crack information can be extracted from strong noise. Gupta et al. [9] investigate the utility of unsupervised machine learning and data visualisation for tracking changes in user activity over time. The goal of semi-supervised and unsupervised image segmentation is to greatly reduce or even eliminate the need for training data, thereby minimizing the burden on clinicians when training the segmentation model. Li et al. [10] present a novelsemi-supervised method for skin lesion segmentation, where the network is optimizedby the weighted combination of a common supervised loss for labeled inputs only anda regularization loss for both labeled and unlabeled data. Pathan et al. [11] proposed a deep clustering architecture and formal image analysis for image segmentation. The main idea is based on an unsupervised learning method, clustering the images of the severity of the disease in the sample of the subject, and then segmenting the image to highlight and outline the area of interest. Feyjie et al. [12] proposed a novel small-scale learning framework for semantic segmentation, in which unlabeled images can be used in each plot.

Recently, the Deep Convolutional Neural Network (DCNN) model segment skin lesions into pixel-level classification problems and achieve remarkable success [13,14,15,16]. No matter from the deep full convolutional neural network (FCN), which earliest uses in image segmentation [17], to UNet [18], which extends the architecture of FCN, and various extension models based on UNet architecture, they all have the contradiction between semantic information and spatial location information. The feature semantic information in the deep layers of the network is richer, but through step-by-step pooling, the spatial resolution of the feature is continuously reduced, and the spatial position information is continuously lost. If the output is directly upsampled to the original input resolution, the segmentation result will be rougher [19]. For dermoscopy images, due to its small grayscale changes and relatively blurred boundaries, the underlying features with rich spatial location information are particularly important for restoring feature spatial resolution.

In recent years, the attention mechanism has been widely used in deep neural networks and has been widely used in many tasks [20,21,22]. Most applications in computer vision and computer graphics involve the concept of image filtering to reduce noise or extract useful image structure. The guided filter [23] is an edge-preserving image filter, which serves as a special extension path that transfers the structural information extracted from the low-level feature map to the high-level feature map. It is effective and efficient in a variety of computer vision and computer graphics applications, including noise reduction, detail smoothing/enhancement, HDR compression, image extinction/feathering, haze removal, and joint upsampling.

In this article, we propose a Channel Spatial Attention-guided network with Densely Connected Convolutional (CSAG and DCCNet) based on deep CNN. The model is equipped with a novel and effective Channel Spatial Fast Attention-guided Filter module (CSFAG) for dermoscopic image segmentation. Compared with the existing attention methods widely used in CNN, the CSFAG module has three advantages. First, we directly impose some constraints on the unknown output by considering the guide image and use spatial attention and channel attention to collect information around the feature map. The calculated attention weight is applied to the guide feature map, so that the model can further capture the correlation of dimensional features, focus on the lesion area, and reduce the influence of noise on the segmentation performance of the model. Secondly, the CSFAG module supports the fusion of multi-resolution features. This module can recover spatial information by filtering low-resolution feature maps and high-resolution feature maps, and merge structural information of various resolution levels to better retain spatial location information. Third, adding the CSFAG module we proposed to the network can greatly improve the performance of lesion segmentation, and the module can be seamlessly integrated into multiple basic segmentation network architectures, with good robustness.

Our specific work content is as follows:

(1) We have designed a novel Channel Spatial Fast Attention-guided Filter (referred to as CSFAG). Through a large number of ablation experiments, it is verified that the CSFAG module is superior to other mainstream attention modules and can be combined with multiple basic segmentation networks, which can effectively improve the segmentation performance of the model.
(2) Embed the proposed CSFAG module into the jump connection of the M-Net segmentation network to form CSAG and DCCNet. In the last step of the CSAG and DCCNet encoding path, densely connected convolutions are used to replace ordinary convolutional layers, and through the idea of “collective knowledge”, the gap between low-level features is bridged and features are effectively aggregated.
(3) On the ISIC-2017 data set, the segmentation performance of CSAG and DCCNet was compared with other latest algorithms. The six indicators of accuracy, sensitivity, specificity, Dice coefficient, Jaccard coefficient, and Matthew correlation coefficient were all achieved very competitive results. CSAG and DCCNet trained in ISIC-2017 was tested on another publicly available data set, PH2, to verify the robustness and cross-dataset performance of our method.

The rest of this paper is arranged as follows: related work is introduced in Section 2. Section 3 introduces the proposed network architecture in detail; experiments and comparison results are explained in Section 4; finally, Section 5 gives our conclusion.

## 2. Work Organization

### 2.1. Semantic Segmentation Model

The method based on full convolutional network (FCN) has made great progress in semantic segmentation. The initial popular depth learning method for semantic segmentation tasks is patch classification, which uses image blocks around the pixel to classify each pixel independently. J. Long et al. [17] first applied the FCN to the end-to-end training of image segmentation, which makes the convolutional neural network can perform dense pixel prediction without a fully connected layer. This method to produce image segmentation maps of any size. Since then, in the field of semantic segmentation, this model is adopted by almost all advanced methods. Except for the fully connected layer, another problem with convolutional neural networks for semantic segmentation is the use of pooling layers. Although the pooling layer expands the receptive field and aggregates the context, it causes the loss of location information.

There are two different structures to solve this problem. The first is the encoder-decoder structure. The encoder gradually reduces the spatial dimension through the pooling layer, and the decoder gradually repairs the details and spatial dimensions of the object through methods such as bilinear interpolation. The encoder and the decoder are usually embedded with a skip connection, which can better help the decoder to repair the details of the target. U-Net [18] is the most commonly used structure in this method. Since then, the UNet-based structure has derived multiple segmentation networks, such as V-Net [24], UNet++ [25], MultiResUNet [26], UNET 3+ [27]. The second method is to use the atrous convolution structure to remove the pooling layer. On the premise of not reducing the spatial dimension, the atrous convolutional layer improves the receptive field index, captures multi-scale context information and maintains the spatial position relative of the feature map, while the pooing will introduce translation invariance. Classical semantic segmentation networks from Deeplab series networks [28,29,30] to PSPNet [31] all use atrous convolution.

### 2.2. Attention Mechanism

As we all know, attention plays an important role in human perception [32,33]. The attention mechanism has been widely used in many tasks. Earlier, the Google Deep Mind team first used the attention mechanism on the RNN model for image description problems [34]. Subsequently, they proposed a model based on the attention mechanism for the recognition of multiple objects in the image [35]. Typical attention models on a single image are residual attention network [36] and squeeze-and-excitation Networks (SENet) [37]. The residual attention network includes two attention components, a stacked network structure composed of multiple attention components, and residual attention learning that combines the residual structure with the attention mechanism. SENet includes a squeeze and excitation block to retain the channel attention introduced for each residual block. PSANet [38] aggregates the contextual information of each location through the predicted attention map. $A^{2}$Net [39] propose a dual attention block, which can collect informative global features from the entire time and space distribution of the image. DANet [40] is based on the self-attention mechanism to capture rich contextual relevance to solve the scene segmentation task, while applying spatial and channel attention to collect information around the feature map. Sanghyun Woo [41] proposed an attention module called CBAM, which can be embedded into classic deep networks to improve model performance. Huisi Wu et al. [42] proposed a deep learning model equipped with a new and efficient adaptive dual attention module (ADAM) to automatically segment skin lesions from dermoscopic images. Most of the above methods only focus on feature information from the two parts of spatial and channel. However, in some recent studies, some scholars use both spatial and temporal domains for object segmentation with self-attention. Hamad et al. [43] proposed dilated causal convolution with multi-head self attention for sensor human activity recognition. The multi-headed self-attention is used to enable the model to focus on important and relevant time steps more than the insignificant time steps from the sequential feature maps during recognition. Hu et al. [44] proposes a hybrid multi-dimensional features fusion structure of spatial and temporal segmentation model for automated thermography defects detection. They design a new attention block to provide spatiotempo-ral attention to focus on semantically meaningful regionsof the volumetric data and recalibrate the feature mapsadaptively, based on the weighted channels.

However, all of the above methods only emphasize the local focus of the pixel relationship or the global focus of the entire image. Due to the different shapes, sizes and colors of lesions; skin types and characteristics; inherent skin characteristics, a single attention mechanism or the existing two-dimensional attention module with dimensional integration still cannot cope with the challenges in skin lesion segmentation. In this work, we combined image filtering with attention, and designed a Channel and Spatial Fast Attention-guided Filter (CSFAG) module to preserve the smooth characteristics of the edges of skin lesions without being subject to gradient inversion The effect of artifacts. The CSFAG module performs well in terms of quality and efficiency.

### 2.3. Dermoscopic Image Segmentation

Earlier, Kawahara, etc. proposed a fully convolutional network based on Alex Net to extract the surface features of melanoma [45]. Subsequently, Long [17] proposed a fully convolutional neural network FCN, which uses a fully convolutional layer instead of a fully connected layer to convert the classification convolutional neural network into a segmentation network. Lequan uses ResNet based on the FCN network structure to segment the lesion area on the ISIC dermoscopic image data set [46]. UNet, proposed by Ronneberger et al. [18], is one of the most popular FCN structures for medical image segmentation. Tschandl P is based on migration learning and uses the LinkNet structure to use the ResNet classification network pre-trained on the ImageNet data set for the coding part of the segmentation. It has achieved a significant performance improvement on the task of segmentation of skin lesions [47]. The DeepLab series of work introduced delitated convolution to reduce the loss of the resolution of the coding part and increase the receptive field. Goyal applied DeepLabV3+ to the task of skin lesions segmentatio [48]. In order to reduce the number of parameters that make the network lightweight, Md. Hasan et al. uses depthwise separable convolution instead of standard convolution, and projects the learned distinguishing features onto the pixel space at different stages of the encoder [49]. However, in clinical practice, due to the complexity of the lesion and the significant increase in the number of dermoscopic images, the lightweight, robustness and ability of the segmentation model to be combined with multiple basic networks are becoming more and more important. In our work, our goal is to develop a segmentation model that is easy to transplant and has good robustness.

## 3. Proposed CSAG and DCCNet Model

**Figure 2:**
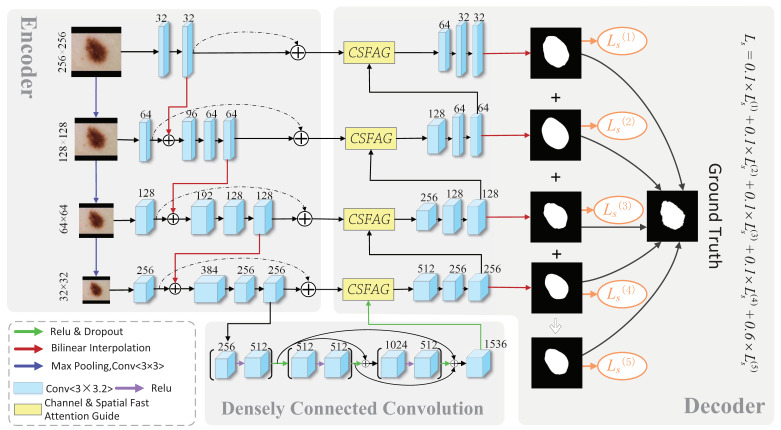
CSAG and DCCNet model architecture.

The CSAG and DCCNet proposed in this paper is an end-to-end multi-label deep network, which consists of multiple novel channel and space fast attention-oriented filter modules (CSFAG), densely connected convolution, multi-scale input layer, UNet and side output layers. In the CSFAG module, the guided filter is combined with the attention module, and a novel attention-oriented filter is designed and embedded in the skip connection of CSAG and DCCNet. In the last step of the CSAG and DCCNet coding path, densely connected convolution is used to replace the ordinary convolutional layer. Using dense connection convolution in the last step of the encoding path can recover spatial information with the help of rich context information, further enriching context information and reducing the difficulty of training. CSAG and DCCNet uses U-Net as the basic network structure, and builds an image pyramid input on the left side of the coding layer to achieve a multi-scale layer of multi-level receiving field fusion; on the right side of the decoder, it introduces a side output layer, average all side output maps as the final prediction map. Simultaneously use the multi-label loss function to update the parameters to train the model The architecture of this model is shown in Figure 2.

### 3.1. CSFAG Module

**Figure 3:**
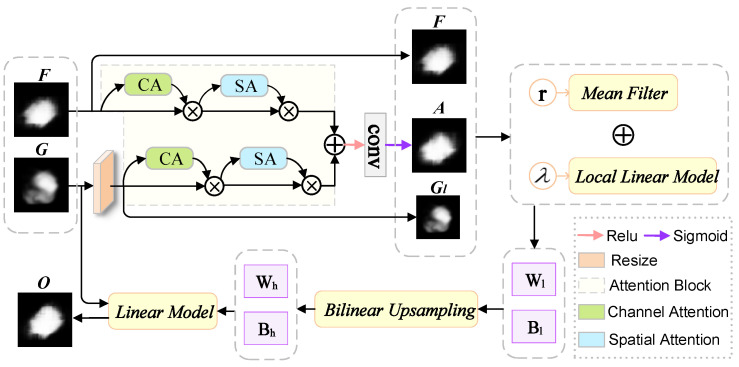
CSFAG model architecture.

The concept of image filtering is widely used in computer vision and computer graphics. Simple linear translation invariant (LTI) filters, such as Gaussian filters, Laplacian filters and Sobel filters, are widely used for image blur/sharpening, edge detection and feature extraction. The kernel of the LTI filter is spatially invariant and has no relation to the image content. Considering the above reasons, we hope to merge other information from the given guide image during the image filtering process so that the image filter can establish a connection with the structural information in the dermoscopic image, in order to better improve the feature extraction ability of the model. We consider using guided images to directly impose some constraints on the output, and use spatial attention and channel attention to collect information around the feature map. Therefore, this article is inspired by the above-mentioned methods and designs a channel and space fast attention-oriented filter (CSFAG) module. The module is mainly composed of two parts, the fast guide filter and the attention module. The model architecture of this module is shown in Figure 3.

The input of the CSFAG module includes the guided feature map *G* and the filtered feature map *F*, and the output is a high-resolution feature map *O*. First, we use bilinear interpolation to scale the guiding feature map *G* to generate a low-resolution feature map $G_{l}$.$G_{l}$ and filtered feature map *F* through attention module to generate attention feature map *A*. We minimize the reconstruction error [50] between $G_{l}$ and *F* to obtain the coefficients $W_{l}$ and $B_{l}$ of the CSFAG module. By bilinearly up-sampling $W_{l}$ and $B_{l}$, we obtain the coefficients $W_{h}$ and $B_{h}$ which match the guiding feature map *G*. Finally, the output *O* of the CSFAG module is obtained through linear transformation.

Specifically, the channel and space fast attention-oriented filter constructs a local square window $\omega_{k}$ with a radius of *r* and a pixel *k* as the center to realize a local linear model. Assuming that $Gl_{i}$ is the pixel of $G_{l}$, the output $Ok_{i}$ of $\omega_{k}$ is obtained by linear transformation:

(1) $$O_{k_{i}}=W_{k}G_{l_{i}}+B_{k},\;\forall i\in \omega_{k}$$

In order to determine the coefficients $W_{k}$ and $B_{k}$, it is necessary to minimize the reconstruction error between the outputs $Ok_{i}$ and the filtered feature $F_{i}$ of all pixels in the window $\omega_{k}$, as shown in Equation (2), where λ is a regularization parameter which controls the smoothness. $A_{i}$ is the attention weight at position *i*, obtained by the attention module:

(2) $$\min_{W,B_{k}}E\left(W_{k},B_{k}\right)=\sum_{i\in \theta k}\left(A_{i}^{2}{\left(W_{k}G_{l}+B_{k}\right)}^{2}+\lambda W_{k}^{2}\right)$$

(3) $$W_{k}=\frac{\frac{1}{\left|\omega \right|}\sum_{i\in \omega k}Gl_{i}F_{i-}\mu_{k}{\bar{F}}_{k}}{\sigma_{k}^{2}+\lambda }$$

(4) $$B_{k}={\bar{F}}_{k}-W_{k}\mu_{k}$$

In Equations (3) and (4), $\mu_{k}$ and $\sigma_{k}$ are the mean and variance of *G* in the window $\omega_{k}$,|ω| is the number of pixels in $\omega_{k}$, and ${\bar{F}}_{k}=\frac{1}{\left|\omega \right|}\sum_{i\in \omega }$ is the average of *F* in $\omega_{k}$.

After calculating the coefficients $W_{k}$ and $B_{k}$ in the window $\omega_{k}$, we get the output $Ok_{i}$ corresponding to each window. Average $Ok_{i}$ obtained from different windows to generate $O_{i}$ which is equal to the average coefficient of all windows overlapping with *i*. As shown in Equation (5),

(5) $$O_{i}=\frac{1}{N_{k}}\sum_{k\in \Omega_{i}}W_{k}G_{i}+\frac{1}{N_{K}}\sum_{k\in \Omega_{i}}B_{k}=W_{l}*G_{l}+B_{l}$$

where $\Omega_{i}$ is the set of all windows including position *i*, and * is element-wise multiplication. After up-sampling $W_{l}$ and $B_{l}$ to obtain $W_{h}$ and $B_{l}$ respectively, the final output is calculated as $O=W_{h}*G+B_{h}$.

The calculation process of the CSFAG module is described in detail in Algorithm 1.

**Figure 4:**
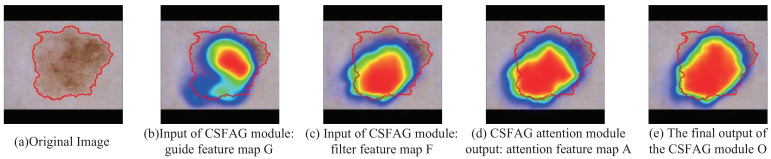
Visual display of CSFAG module input and output (A dermoscopy image of melanoma is a case, the red line is ground truth).

In order to further demonstrate that the CSFAG module can help the model generate clearer boundaries, highlight the lesion area and reduce background influence, We randomly selected a melanoma dermoscopy image in the test set of the ISIC2017 data set for visual analysis. We select the CSFAG module on the jump connection in the fourth layer of the CSAG and DCCNet model as the test module. For showing the role of the CSFAG module more clearly, we perform three convolution operations on the input of the CSFAG module, the output of the intermediate attention module and the final output of the CSFAG module to reduce the dimensionality. Then use bilinear interpolation to restore the feature size to 256×256. We have drawn a CAM heat map to more clearly show the changes produced by the CSFAG feature map. As shown in Figure 4, we can clearly see from the CAM heat map, after the attention module in the CSFAG module, the features are more focused on the lesion area. In particular, the final output results generated after the fast guide filter, the model further aggregates the characteristics of the space and the channel, enhances the discriminative ability of the network, and makes the network good use of the given characteristics.
**Algorithm 1** Channel Spatial Fast Attention-guided Filter
**Input:** the guided feature map *G* and the filtered feature map *F*, parameters are *r* and λ**Output:** high-resolution feature map *O*
1:$G_{l}$ = $f_{subsample}$(G)2:$F_{CA}$ = $f_{ChannelAttention}$(F), $F_{SA}$ = $f_{SpatialAttention}$$\left(F_{CA}\right)$$G_{CA}$ = $f_{ChannelAttention}$$\left(G_{l}\right)$, $G_{SA}$ = $f_{SpatialAttention}$$\left(G_{CA}\right)$3:$A_{0}$= $F_{SA}$+$G_{SA}$, $A_{1}$ = $F_{Relu}$$\left(A_{0}\right)$, $A_{2}$ = $F_{convolution}$$\left(A_{1}\right)$, *A* = $F_{sigmod}$$\left(A_{2}\right)$4:$\omega_{k}$ = a local square window with a radius of *r* and a pixel *k* as the ccenter5:$\mu_{k}=\frac{1}{N}\sum_{i=0}^{N}G_{l},\forall i\in \omega_{k}$6:$\sigma_{k}^{2}=\frac{1}{N}\sum_{i=0}^{N}{\left(i-\mu \right)}^{2}G_{l},\forall i\in \omega_{k}$7:${\bar{F}}_{k}=\frac{1}{\left|\omega \right|}\sum_{i=0}^{N}F,\forall i\in \omega_{k}$8:$W_{k}=\frac{\frac{1}{\left|\omega \right|}\sum_{i\in \omega k}Gl_{i}F_{i-}\mu_{k}{\bar{F}}_{k}}{\sigma_{k}^{2}+\lambda }$9:$\min_{Wk,Bk}E\left(W_{k},B_{k}\right)=\sum_{i\in \omega k}\left(A_{i}^{2}{\left(W_{k}G_{l}+B_{i}\right)}^{2}+\lambda W_{k}^{2}\right)$10:$O_{k_{i}}=W_{k}G_{l_{i}}+B_{k},\;\forall i\in \omega_{k}$11:$W_{l}=\frac{1}{N_{k}}\sum_{k\in \Omega i}W_{k}$,$B_{l}=\frac{1}{N_{k}}\sum_{k\in \Omega i}B_{k}$12:$O_{i}=W_{l}*G_{l}+B_{l},\forall i\in G$13:$W_{h}$ = $f_{upsample}$$\left(W_{l}\right)$, $B_{h}$ = $f_{upsample}\left(B_{l}\right)$14:$O=W_{h}*G+B_{h}$15:** return** *O*


#### Channel and Spatial Attention Learning

**Figure 5:**
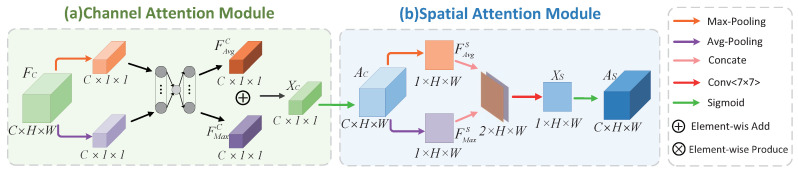
Attention module; (**a**) channel attention submodule; (**b**) spatial attention submodule.

The attention mechanism on the channel has been proposed in Hu et al.’s SENet [37], and it has been verified that it can improve network performance. The channel attention module and the spatial attention module [41] are shown in Figure 5. The Channel Attention Module compresses the feature map in the spatial dimension to obtain a one-dimensional vector before performing operations. As shown in Figure 5a, the input feature map $F_{C}\in R^{C\times H\times W}$ is passed through global max pooling and global average pooling based on width and height respectively, and then passed through a multilayer perceptron (MLP). The MLP output feature maps $F_{Avg}^{C}\in R^{C\times 1\times 1}$ and $F_{Max}^{C}\in R^{C\times 1\times 1}$ are summed element-by-element, so that channel attention maps Xc can be generated, as shown in Equation (6). After the sigmoid activation operation, the final channel attention feature map d is generated, as shown in Equation (7). The channel attention featuremap and input featuremap are subjected to elementwise multiplication operations to generate the input features required by the spatial attention module.

(6) $$Xc=F_{Avg}^{C}⊕F_{Max}^{C}\;,\;Xc\in R^{C\times 1\times 1}$$

where ⊕ represents addition element by element.

(7) $$A_{c}=\overset{c}{\underset{i=0}{⋃}}\frac{1}{1+\exp\left(-X_{i,1,1}\right)}\;,\;A_{c}\in R^{C\times 1\times 1}$$

where *C* is the number of channels, $X_{i,1,1}$ is the element with coordinate (i,1,1), and ⋃ is element-by-element contacting.

The Spatial Attention Module compresses the channel, and performs average pooling and maximum pooling in the channel dimensions. As shown in Figure 5b, the feature map $A_{c}\in R^{C\times H\times W}$ output by the channel attention module is used as the input feature map of this module. First, the average pooling and maximum pooling operations along the channel axis are used to obtain two two-dimensional feature maps $F_{Avg\;}^{S}$ and $F_{Max}^{S}$ (the specific process is shown in Equations (9) and (10)), and then concat the two results based on the channel. The feature map of the merged 2 channels is subjected to a convolution operation to reduce the dimension to 1 channel to generate a spatial attention map Xs, as shown in Equation (8), where *W* and *b* represent MLP weight and MLP biase respectively. Cat(.) means concatenate. Conv(.) means convolution operation.

(8) $$Xs=Conv(Cat\left(F_{Avg}^{S};F_{\mathrm{Max}\;}\right)W+b,Xs\in R^{1\times H\times W})\;$$

(9) $$F_{Avg}^{S}\left(x,y\right)=\frac{1}{C}\sum_{i=1}^{c}X_{i,x,y},\;F_{Avg}^{S}\in R^{1\times H\times W}$$

(10) $$F_{Max}^{S}\left(x,y\right)={\mathrm{Max}}_{i=0}\left(X_{i,x,y}\right),\;F_{Max}^{S}\in R^{1\;\times H\times W}$$

Then generate spatial attention feature $A_{S}$ through sigmoid, as shown in Equation (11), and finally multiply the feature and the input feature of the module to obtain the final generated feature.

(11) $$A_{S}=\overset{H}{\underset{m}{⋃}}\overset{w}{\underset{n}{⋃}}\frac{1}{1+\exp\left(-Xs_{m,n}\right)}\;,\;A_{s}\in R^{1\times H\times W}$$

where *m*, *n* represent *m*th position and *n*th position respectively. ⋃ represents the contact element by element.

### 3.2. Densely Connected Convolution Module

**Figure 6:**
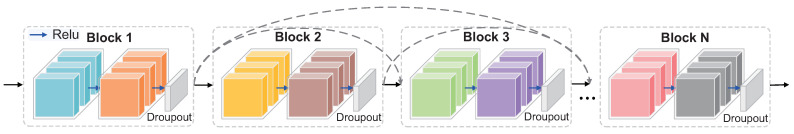
Densely connected convolution module.

The densely connected convolution module [51] is composed of multiple dense blocks, and each dense block performs two convolution operations. The structure of densely connected convolution is shown in Figure 6. The distinctive feature of the densely connected convolution module is that the input of each block is the concatenation of all feature maps generated by all previous blocks, and each layer performs a series of continuous transformations. The idea of densely connected convolution has some advantages over conventional convolution. First, it helps the network learn diversified functional characteristics, rather than redundant functions. In addition, this idea allows information to flow through the network and reuse functions to increase the representativeness of the network. Dense connectivity ensures the maximum information path between layers by connecting all layers. The output features of all convolutional layers in the dense block are connected in series along the channel axis. Let $X_{i-1}$ and $X_{i}$ be the input and output of the i−th tightly connected dense block, respectively. The output $X_{i}$ of the i−th dense block $F_{i}$ can be obtained in the following manner.

(12) $$X_{i}=F_{i}\left(\left[X_{i-1},X_{i-2},\ldots ,X_{0}\right]\right)$$

where [.] represents the element series along the channel axis, and the input $X_{0}$ is the initial element $f_{1}$. Because the input of the convolutional layer is repeatedly cascaded, the number of channels used for the input of the next convolutional layer increases with the growth rate *n*.

## 4. Results and Discussion

### 4.1. Data Set and Processing

#### 4.1.1. Data Set

The data used in this article were from the ISIC2017 [52] challenge data set released by the International Skin Imaging Collaboration (ISIC) and the PH2 [53] data set provided by the Pedro Hispano Dermatology Department of the Hospital and the Tecnico Lisboa University of Porto Research Group in Matosinhos, Portugal. The ISIC2017 data set provided 2750 dermoscopic images in RBG format, of which 2000 images were used in the training phase, 600 images were used in the Test Phase, and 150 images were used in the Validation Phase. The PH2 data set contained 200 8-bit RGB dermatoscope images with a fixed size of 768×560 pixels. All images provided lesion boundaries given by professional clinicians. Table 1 summarizes the distribution of the two data sets.

Ne, Me and SK stand for Benign Nevus, Melanoma or Seborrheic Keratosis, respectively.

#### 4.1.2. Processing

**Figure 7:**
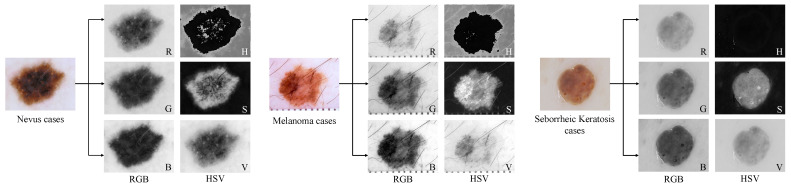
Visual description of the input dermoscopy image in different color spaces.

We combined 150 test data and 2000 training data as training data for the network. In order to train our proposed network structure more conveniently, we scaled the width of images of different sizes to 256 px according to the aspect ratio, and then filled the black borders on the top and bottom of the image to increase the height to 256 px. In order to better allow the network to learn the brightness, tone, and vividness of the dermatoscope image, we converted the image in RGB format to HSV format. As shown in Figure 7, the color components of R, G, and B in the RGB image were all related to the amount of light irradiated to the object. Therefore, the image description based on these components made it difficult to distinguish the object. Unlike RGB, HSV separates brightness or image intensity from chromaticity or color information and is more stable to changes in external lighting. HSV images can detect objects with specific colors and reduce the influence of light intensity from the outside. Therefore, we used HSV images as an effective supplement to training data to train the segmentation network. At the same time, the two formats of images were rotated horizontally, vertically, horizontally and vertically to expand the number of training images, and finally 17,200 training images were generated.

### 4.2. Experimental Setup

#### 4.2.1. Evaluation Metrics

We used Sensitivity (SEN), Specificity (SPE), Accuracy (ACC), Jaccard (JAC), Dice Coefficient (DIC) and Matthew Correlation Coefficient (MCC) to more accurately judge the segmentation performance of CSAG and DCCNet proposed in this paper. Among them, SEN, SPE, and ACC are common statistical measures used to judge the performance of binary classification. JAC and DIC are used to evaluate the similarity between the segmentation results and ground truth. MCC is the correlation coefficient between the prediction result and ground truth. The above evaluation indicators are directly calculated from the confusion matrix. The calculation method of these six evaluation indicators refers to Equations (13)–(18), where TP represents the correct segmentation of skin lesion pixels, and FN is the wrong segmentation of skin lesion pixels. If the segmentation of non-lesion pixels is correctly classified as non-lesion, it is regarded as TN. Otherwise, they are FP.

(13) $$Sensitivity=\frac{TP}{TP+FN}$$

(14) $$Specificity=\frac{TN}{FP+TN}$$

(15) $$Accuracy=\frac{TP+TN}{TP+FN+TN+FP}$$

(16) $$JAC=\frac{TP}{TP+FN+FP}$$

(17) $$DIC=\frac{2\times TP}{2\times TP+FP+FN}$$

(18) $$MCC=\frac{TP\cdot TN-FP\cdot FN}{\sqrt{\left(TP+FP)(TP+FN)(TN+FP)(TN+FN\right)}}$$

#### 4.2.2. Implementation Details

All experiments in this article were run on Ubuntu 16.04 system, performed on a workstation equipped with Intel(R) Xeon(R) Gold 5218 CPU 2.30 GHz, NVIDIA Quadro RTX 6000 (24 G). Use Python 3.6 and Pytorch 1.0.0 deep learning framework for programming. CSAG and DCCNet used stochastic gradient descent with momentum, the momentum parameter was 0.9, the weight decay coefficient was 5 × 10${}^{-4}$, the initial learning rate was 0.1, the exponential decay was 10% every 50 epochs, and the mini-batch size was 16. We used the Softmax function for final classification. The radius *r* of the local square window constructed in the CSFAG module was 4, and the regularization parameter λ was set to 0.001.

### 4.3. Ablation Analysis

#### 4.3.1. Discussion on the Number of Densely Connected Blocks at the Bottom

In the last step of the CSAG and DCCNet encoding path proposed in this article, densely connected convolution was used to replace the ordinary convolutional layer. However, setting several densely connected blocks could achieve the best segmentation results, which is a problem worth discussing. Therefore, this article used 600 dermoscopy images on the ISIC-2017 data set for testing, and the distribution of the 600 test images is shown in Table 2. We used the control variable method. In the skip connection part of CSAG and DCCNet, the CSFAG module was still embedded, and only the number of dense connection blocks *D* used in the last step of the encoding path changed. We set *D* = 1, 2, 3, and 4 respectively. When *D* = 1, there was no densely connected convolution block in this layer, which was similar to the last coding layer of the standard U-Net.

The specific experimental results are shown in Table 2. When the number of densely connected blocks was set to 3, the best results were achieved on ACC, SPE, DIC, JAC, and MCC. When *D* = 4, all indicators fell. In subsequent experiments, three densely connected blocks were set.

#### 4.3.2. Structure Ablation

The CSAG and DCCNet proposed in this paper uses U-Net as the basic network structure, and builds an image pyramid input on the left side of the coding layer to achieve a multi-scale layer of multi-level receiving field fusion; on the right side of the decoder, introduces a side output layer, average all side output maps as the final prediction map. This structure also constitutes M-Net [54]. In addition, the use of densely connected convolution and CSFAG module are also key factors for the CSAG and DCCNet model to show excellent performance in skin lesion segmentation. In this regard, we designed a set of ablation experiments, taking the UNet network structure as the baseline model, and adding image pyramid input, side output layers and multi-label loss functions to this basic structure to verify that these methods can improve the segmentation of dermoscopic images. In the last step of the MNet encoding path, densely connected convolutions are used instead of ordinary convolutional layers. We named them M-Net+Dense Convolutions. The CSFAG module is embedded on the MNet skip connection, and we named it M-Net+CSFAG. Experiments have further proved that these two methods are effective. The specific segmentation performance indicators are shown in Table 3 and Table 4. The data in all tables were tested using different models on the 600 test set images in the ISIC2017 data set, calculating the various indicators of each test image, and averaging the various indicators of the 600 test images. The results obtained for Nevus cases, Melanoma cases, SK cases, and overall were all strictly trained for 200 epochs.

From the results in Table 3 and Table 4, we can see that whether it was in Benign Nevus, Melanoma and Seborrheic Keratosis lesions, MNet achieved greater improvement in various indicators compared with UNet, which verified Pyramid input, side output and multi-label loss function methods were helpful to improve the performance of dermoscopy image segmentation. From the results of M-Net+Dense Convolutions and M-Net+CSFAG, compared with MNet, the four key indicators of ACC, DIC, MCC, and JAC all achieved certain improvements. This further proves that these two methods are effective. We applied Dense Convolutions and CSFAG to MNet together to design the CSAG and DCCNet proposed in this article. From the specific segmentation results, especially in the segmentation of melanoma lesions and seborrheic keratosis, very competitive results could be obtained.

**Figure 8:**
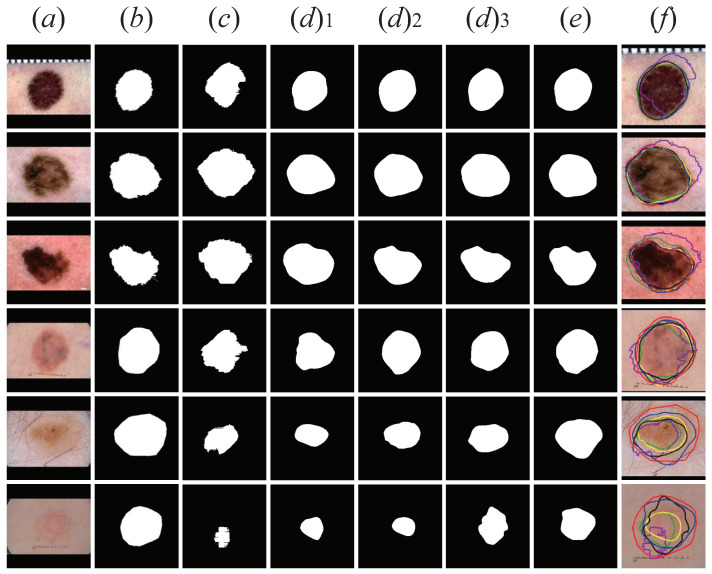
Visualized results of structural ablation experiments. The first two lines are Benign Nevus, the middle two lines are Melanoma lesions, and the last two lines are Seborrheic Keratosis lesions. (**a**) Original image; (**b**) the ground truth; (**c**) U-Net; (**d**) 1. M-Net; (**d**) 2. M-Net+Dense Convolutions; (**d**) 3. M-Net+ CSFAG; (**e**) CSAG and DCCNet model; (**f**) ground truth (red) and U-Net+ (purple), MNet (green), M-Net+Dense Convolutions (yellow), M-Net+CSFAG (black) and ours (blue) segmentation results comparison chart. All pictures are preprocessed.

As presented in Figure 8, we visually display the segmentation results of UNet, MNet, M-Net+Dense Convolutions, M-Net+CSFAG, and CSAG and DCCNet proposed in this section. For Figure 8f, we zoomed in on the lesion area on the image to visually show the comparison between the five models verified in the structure ablation experiment and the ground truth segmentation results. It can be clearly seen in the figure that, compared with UNet, the segmentation result of MNet had clearer edges and more concentrated recognition of the lesion area. For M-Net+Dense Convolutions, M-Net+CSFAG and ours, compared with MNet, the segmentation results on moles and melanoma lesions had little difference. We believe that for dermoscopic images of moles and melanoma lesions, due to the large difference in the front background and the relatively concentrated lesion area, a simpler model could obtain better segmentation results. However, for the small difference between the front background and the blurred boundary of the lesion area, similar to the dermoscopic image of the last two rows in Figure 8, it was difficult to obtain a more ideal segmentation result using a more basic segmentation network structure such as UNet or MNet. Therefore, CSAG and DCCNet was designed in this paper. From the segmentation results of the last two rows in Figure 8, CSAG and DCCNet could identify larger lesion areas for images with small differences in the front background and blurred boundaries of the lesion area, the segmentation result obtained was closer to the ground truth.

#### 4.3.3. Attention Module Ablation

The Channel Spatial Fast Attention-guided Filter (CSFAG) module designed in this paper is the main reason for the improvement of segmentation performance. In order to verify the effectiveness of the CSFAG module and verify that it had better feature aggregation performance than other attention modules, we designed a set of ablation experiments. We use M-Net+Dense Convolutions in the structural ablation part as the Baseline, and only changed the attention module embedded in the jump connection to verify the advanced nature of the CSFAG module proposed in this article. Three classic, lightweight, and general attention modules were selected for comparison with the CSFAG module. The first was the Squeeze-and-Excitation module in SE-Net [37]. The SE module performs attention or gating operations on the channel dimension to drive the model to pay more attention to the channel features with the most information, while suppressing those unimportant channel features. The second attention module is the Feature Pyramid Attention (FPA) module proposed by Li and Xiong et al. [55]. This module performs attention operations on pixels, adopts the idea of global pooling of PSPnet [31], and adds the result of pooling to the result of convolution with attention. The last attention module is the CBAM module proposed by Sanghyun Woo [41]. This module combines the attention mechanism of spatial and channel, and learned how to effectively emphasize or compress and extract intermediate features, so that the model pays more attention to the target object itself.

Table 5 and Table 6 show the segmentation performance of the model formed by the combination of SE Block, FPA, CBAM and Baseline and CSAG and DCCNet (ours) on three skin lesions. From the perspective of various indicators, the segmentation performance of the network structure with the added attention module was greatly improved compared with the baseline. For CSAG and DCCNet, especially in the three key indicators of DIC, JAC, and MCC, CSAG and DCCNet has achieved good results. We think the reasons why CSAG and DCCNet could obtain higher results are considered as follows. SE Block only studied the channel attention mechanism of feature maps, and ignored the information in the spatial dimension. Although the FPA module used the pyramid structure to expand the range of the receptive field, it lost the pixel-level positioning information, which had a greater impact on the dermoscopic image segmentation. CBAM could learn what to pay attention to and where to pay attention in the channel and spatial dimensions, but it is difficult to integrate the structural information contained in low-resolution feature maps and high-resolution feature maps, and retains less edge information. In addition, for the parameters of each model, SE Block only paid attention to the characteristics at the channel level, making the model parameters smaller. The FPA module uses 5×5 and 7×7 large convolution kernels, resulting in a large amount of model parameters. CBAM applies attention to both channel and spatial dimensions at the same time. Compared with SE Block, the amount of CBAM module parameters also increased. However, the CSFAG module proposed in this paper used the CBAM module as the attention module and combined the guide filter, so the parameter amount was slightly higher than the CBAM module parameter amount.

**Figure 9:**
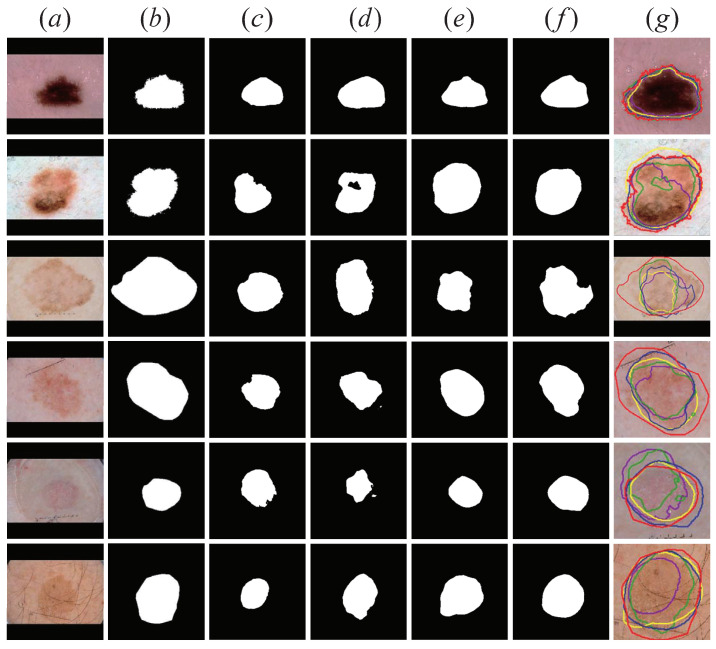
Visualization results of the attention module ablation. The first two lines are Benign Nevus, the middle two lines are Melanoma lesions, and the last two lines are Seborrheic Keratosis lesions. (**a**) Original skin lesion image; (**b**) Ground truth corresponding to the lesion; (**c**) SE Block+Baseline model lesion segmentation results; (**d**) FPA+Baseline model lesion segmentation results; (**e**) CBAM+Baseline model lesion segmentation Results; (**f**) CSAG and DCCNet (ours) model lesion segmentation results; (**g**) ground truth (red) and SEnet+Baseline (purple), FPA+Baseline (green), CBAM+Baseline (yellow) and ours (blue) segmentation Results comparison chart. All pictures are preprocessed.

For the problems of the above several attention modules, in order to further improve the segmentation performance of the model, we designed the CSFAG module. In Figure 9, we show the segmentation results of the four network structures for ablation experiments in this section. From Figure 9, we found that compared to the other three attention modules, the segmentation results obtained by CSAG and DCCNet (ours) were closer to ground truth. Especially for dermoscopic images with relatively blurry lesions, CSAG and DCCNet (ours) could segment larger lesions and get better segmentation results. In order to compare the segmentation results intuitively, we marked the segmented edge contours with lines of different colors and superimposed them on the original image, and enlarged the lesion area, as shown in Figure 9g. Obviously, the blue line representing CSAG and DCCNet (ours) was closer to the red line representing ground truth than the other three modules, which proves that the CSFAG module could achieve better segmentation performance on the baseline model.

**Figure 10:**
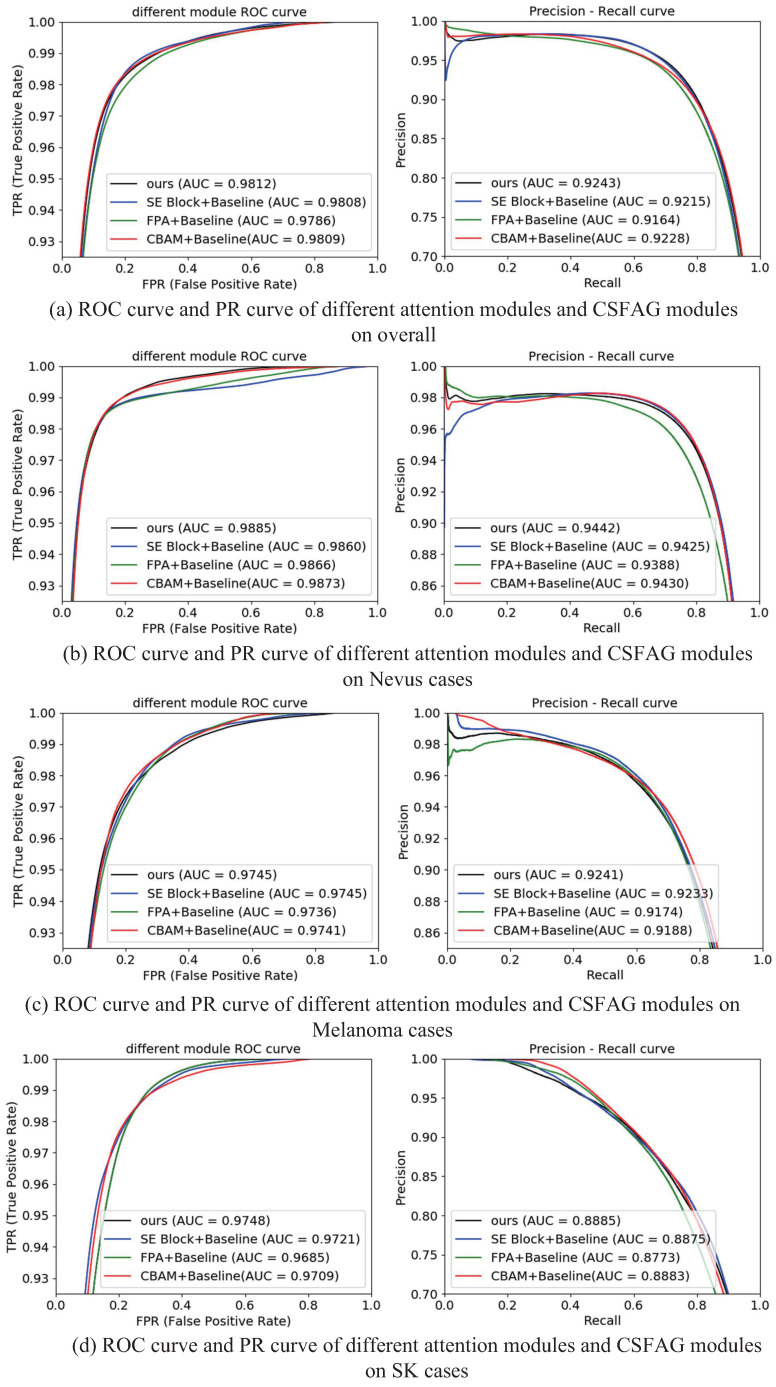
Visualized results of ROC curve and PR curve.

In order to further illustrate the superiority of the CSFAG module, we drew the ROC curve and PR curve on the baseline-based SE block, FPA, CBAM model and CSAG and DCCNet (ours) respectively on overall, Nevus cases, Melanoma cases, and SK cases. The ROC curve gave information between false positive pixels and true positive pixels in the form of scores based on the threshold change on the probability map. When the ratio between the positive sample and the negative sample was large, the PR curve could better reflect the true performance of the classification. The difference from the upper left convex of the ROC curve was that the PR curve had an upper right convex effect. As shown in Figure 10, the areas of our ROC curve and PR curve were larger than other attention modules. This further showed that the CSFAG module had better performance than other attention modules, and better segmentation of background information and skin lesion area information.

#### 4.3.4. Robustness Test of CSFAG Module

In order to verify that the CSFAG module proposed in this paper had good robustness and prove that it can be combined with multiple basic segmentation networks, we designed this set of experiments. We used U-Net [18], SegNet [56] and M-Net [54] three basic segmentation networks as the backbone network, embed the CSFAG module into the skip connection, named U-Net+CSFAG, SegNet+CSFAG and M-Net+CSFAG. Table 7 and Table 8 summarize the comparison of the segmentation performance of the three basic architectures and the new model combined with the CSFAG module and the proposed CSAG and DCCNet (ours). From Table 7 and Table 8, we can see that although the new network formed after adding the CSFAG module had a large increase in the amount of model parameters, the new network was higher than the basic network in six indicators, which showed that the CSFAG module could significantly improve the segmentation performance of the network and is easy to transplant, and has good robustness.

The results of UNet and SegNet in Table 7 and Table 8 were slightly lower than those of Al-Masni et al. [57] in Table 9 and Table 10. We considered the differences caused by different parameter settings and training methods.Al-Masni et al. used the weight parameters of the model trained on VGG-16 on the ImageNet dataset as the initial weight of SegNet, and only fine-tuned the weight of the segmentation network. They used Theano and Keras for programming, select the AdamOptimizer optimization algorithm, set the batch to 20, and used NVIDIA GeForce GTX 1080 (16 G) GPU for training. However, when we reproduced U-Net and SegNet, we set random initial weights, performed on a workstation equipped with Intel(R) Xeon(R) Gold 5218 CPU 2.30 GHz, NVIDIA Quadro RTX 6000 (24 G). We used Python 3.6 and Pytorch 1.0.0 deep learning framework for programming. Using the stochastic gradient descent method with momentum, the momentum parameter was 0.9, and the weight attenuation coefficient was 5 × 10^−4^.

**Figure 11:**
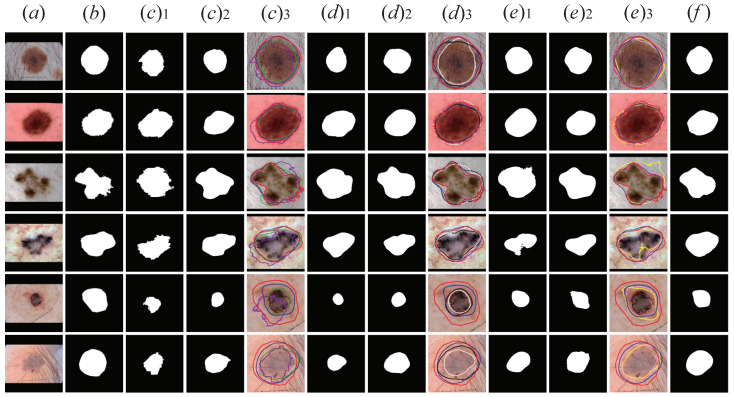
Visualization results of the robustness test of the CSAG module on the ISIC 2017 data set. The first two lines are Benign Nevus, the middle two lines are Melanoma lesions, and the last two lines are Seborrheic Keratosis lesions. (**a**) Original image; (**b**) the ground truth; (**c**) 1. U-Net; (**c**) 2. U-Net+CSFAG; (**c**) 3. Comparison of segmentation results ground truth (red), U-Net (purple), U-Net+CSFAG (green) and ours (blue); (**d**) 1. SegNet; (**d**) 2. SegNet+CSFAG; (**d**) 3. Comparison of segmentation results of ground truth (red) and SegNet (white), SegNet+CSFAG (black) and ours (blue) (**e**) 1. M-Net; (**e**) 2. M-Net+CSFAG; (**e**) 3. Comparison of segmentation results of ground truth (red) and M-Net (yellow), M-Net+CSFAG (pink) and ours (blue) (**f**) CSAG and DCCNet (ours) model. All pictures are preprocessed.

As shown in Figure 11, in order to visually display the improvement of segmentation performance brought by the CSFAG module, we visually display the segmentation results of U-Net, SegNet, M-Net and the new attention-guided network they generate and CSAG and DCCNet. For (c3), (d3), (e3) in Figure 11, we use the red line to represent the ground truth, and the blue line to represent the segmentation result of ours. We zoomed in on the lesion area on the picture to visualize the comparison between the three models of the CSFAG module and the ground truth segmentation results. (c3) shows the comparison of U-Net and U-Net+CSFAG segmentation results. (d3) shows the comparison of SegNet and SegNet+CSFAG segmentation results. (e3) shows the comparison of M-Net and M-Net+CSFAG segmentation results Contrast. It can be clearly seen in the figure that the three backbone networks were compared with the new attention-guided filter network. The new attention-guided filter network could significantly improve the segmentation performance and make the segmentation boundary more accurate. Especially in the dermoscopic image segmentation with small difference between the front and background, the network after adding the CSFAG module could distinguish larger lesion areas. It can be seen that adding the CSFAG module that we proposed to the network could greatly improve the performance of lesion segmentation. The module could be seamlessly integrated into multiple basic segmentation network architectures, and had good robustness.

#### 4.3.5. Comparing with Existing Technology by Lesion Type

Table 9 and Table 10 compare the CSAG and DCCNet proposed in this paper with the latest methods proposed by Al-Masni et al. [57] and Goyal et al. [58]. From the results shown in Table 9 and Table 10, compared with other models, CSAG and DCCNet achieved higher results in all indicators in the segmentation of three different types of skin lesions, especially in ACC, The four key indicators of DIC, JAC and MCC achieved the very competitive results. This proves that our proposed CSAG and DCCNet could produce more accurate segmentation boundaries, especially in the segmentation of melanoma lesions and seborrheic keratosis images. Therefore, we believe that the CSAG and DCCNet proposed in this paper was an effective supplement to the dermoscopic image segmentation method.

Since there are few papers on calculating segmentation performance according to the types of skin lesions, there is a lack of comparable data. Most studies do not divide the test set according to the type of lesion, but evaluate the segmentation performance of all dermoscopic images in the test set together. In order to further compare with existing dermoscopic image segmentation methods, we have compiled the results in Table 11. From the results shown in Table 11, CSAG and DCCNet achieved high results in the two indicators of ACC and SPE, and also achieved more competitive results in the two key indicators of DIC and JAC. This further proved that the CSAG and DCCNet proposed in this paper was an effective supplement to the existing dermoscopic image segmentation methods.

#### 4.3.6. Results on the PH2 Data Set

**Figure 12:**
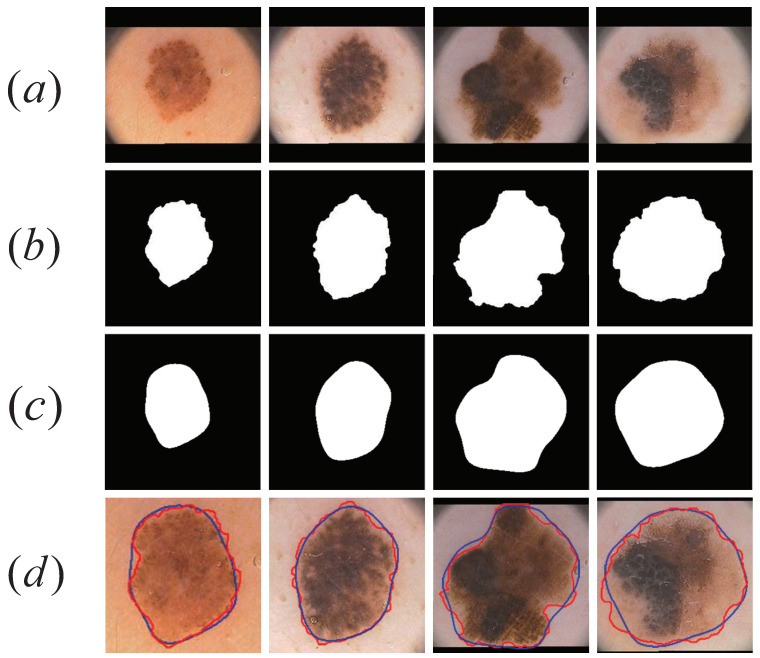
Visualization results for the PH2 data set. (**a**) Original skin lesion image; (**b**) ground truth corresponding to the lesion. (**c**) CSARM-CNN model lesion segmentation results; (**d**) ground truth (red) and segmentation results (blue) comparison chart.

We used the independent dermoscopic image data set PH2 to verify the cross-data performance of our proposed network. We used the model trained on the ISIC2017 dataset to test the segmentation performance on the PH2 dataset. As shown in Table 12 and Table 13, in the segmentation of Benign Nevus and melanoma, our proposed model obtained competitive results. This showed that our proposed model had good robustness and cross-data performance. Figure 12 shows the CSAG and DCCNet segmentation results. The first two columns are moles and the last two columns are melanomas.

## 5. Conclusions

In this article, we propose and implement a novel and robust skin lesion segmentation depth model called CSAG and DCCNet. In the last step of the encoding path, the model uses densely connected convolution instead of ordinary convolutional layers. In order to achieve better information fusion, highlight the foreground and reduce the impact of the background, we designed a novel attention-guided filter module, Channel Spatial Fast Attention-guided Filter (CSFAG for short), and embedded it in the CSAG and DCCNet segmentation network Jumping connection. Secondly, the model uses U-Net as the basic network structure, and builds an image pyramid input layer on the left side of the coding layer; on the right side of the decoder, introduces a side output layer, and averages all side output images as the final prediction image. In order to verify its effect, we evaluated the model using two publicly available datasets (ISIC-2017 Challenge and PH2 dataset). The results show that both densely connected convolution and CSFAG modules can improve the segmentation performance of the network, and the combination of them to form CSAG and DCCNet is better than some of the latest algorithms for skin lesion segmentation. Through a large number of ablation experiments, we have verified that the CSFAG module is superior to other mainstream attention modules and can be combined with multiple basic segmentation networks, effectively improving the segmentation performance of the model. CSAG and DCCNet trained on ISIC-2017 data set was tested on another publicly available data set PH2 data set to verify the robustness and cross-data set performance of our method. In the future, we believe that more interference in dermoscopic images is a key factor affecting segmentation performance, data purification on it is a very effective work. We look forward to the combination of the new dermoscopic image preprocessing method and the proposed model to get a better segmentation model, and apply the model to other medical images to prove the robustness of the method.

## Figures and Tables

**Table 1:** The distribution of The ISIC Challenge 2017 data set and PH2 datasets.

Dataset	ISIC 2017				PH2			
	Me	SK	Ne	Total	Me	SK	Ne	Total
Training data	404	296	1450	2150	-	-	-	-
Test data	117	90	393	600	40	-	160	200

**Table 2:** Determination of the number*D* of densely connected blocks on the ISIC-2017 data set. Bold data indicates that the value is the maximum value in this indicator.

Method	Overall					
	ACC	SEN	SPE	DIC	JAC	MCC
*D* = 1	95.04	**87.58**	96.40	84.96	75.30	81.49
*D* = 2	95.21	87.51	96.99	85.90	77.91	82.51
*D* = 3	**95.94**	87.03	**99.35**	**86.97**	**78.85**	**83.93**
*D* = 4	95.61	86.50	97.46	86.35	77.89	83.50

**Table 3:** ISIC-2017 data set, performance comparison of U-Net, M-Net, M-Net+DC (Dense Convolutions), M-Net+CSFAG and CSAG and DCCNet (ours) in sensitivity, specificity and accuracy. Bold data indicates that the value is the maximum value in this indicator.

Method	Params	Nevus Cases			Melanoma Cases			SK Cases			Overall		
		SEN	SPE	ACC	SEN	SPE	ACC	SEN	SPE	ACC	SEN	SPE	ACC
U-Net	8.6M	81.80	97.53	93.83	70.31	98.08	89.62	57.84	98.34	89.07	74.53	97.59	92.32
M-Net	10.92M	85.96	97.31	96.05	78.60	97.58	92.17	72.60	98.32	91.69	83.22	98.32	94.58
M-Net+DC	13.79M	87.63	97.98	96.13	79.62	97.97	93.34	68.80	99.60	92.89	84.46	99.15	95.10
M-Net+CSFAG	24.89M	**89.92**	97.63	96.82	79.40	97.20	93.24	69.94	98.02	93.39	86.54	97.41	95.85
Ours	28.74M	89.85	**99.43**	**97.46**	**80.23**	**99.51**	**94.70**	**80.24**	**99.70**	**94.99**	**87.03**	**99.35**	**95.94**

**Table 4:** On the ISIC-2017 data set, performance comparison of U-Net, M-Net, M-Net+DC (Dense Convolutions), M-Net+CSFAG and CSAG and DCCNet (ours) in Jaccard coefficient, Dice coefficient and Matthew correlation coefficient. Bold data indicates that the value is the maximum value in this indicator.

Method	Params	Nevus Cases			Melanoma Cases			SK Cases			Overall		
		DIC	JAC	MCC	DIC	JAC	MCC	DIC	JAC	MCC	DIC	JAC	MCC
U-Net	8.6M	78.71	64.89	75.12	73.16	57.68	66.73	64.39	47.49	58.55	74.32	59.13	69.64
M-Net	10.92M	86.89	77.34	84.80	81.72	69.20	78.36	74.32	59.13	71.56	83.68	74.08	81.30
M-Net+DC	13.79M	87.14	78.16	85.56	83.31	72.13	77.11	78.19	62.80	73.85	84.74	76.65	81.78
M-Net+CSFAG	24.89M	88.46	79.31	86.68	83.70	72.71	**79.83**	79.16	65.51	75.20	85.63	76.86	83.20
Ours	28.74M	**90.65**	**82.90**	**89.19**	**84.24**	**73.68**	78.10	**82.14**	**72.78**	**77.56**	**86.97**	**78.85**	**83.93**

**Table 5:** Performance evaluation of sensitivity, specificity, and accuracy performance of different attention modules and CSFAG modules on the ISIC-2017 data set using baseline as the basic structure. Bold data indicates that the value is the maximum value in this indicator.

Method	Params	Nevus Cases			Melanoma Cases			SK Cases			Overall		
		SEN	SPE	ACC	SEN	SPE	ACC	SEN	SPE	ACC	SEN	SPE	ACC
SE Block+Baseline	26.62M	**90.25**	97.05	96.89	78.77	98.68	93.41	78.23	98.04	94.01	85.91	97.97	95.44
FPA+Baseline	32.08M	88.26	98.67	96.21	76.12	98.22	93.57	77.20	98.12	93.45	82.29	98.15	95.35
CBAM+Baseline	27.40M	89.01	98.25	97.06	76.05	97.75	93.59	**81.69**	96.68	94.16	82.76	97.70	**95.99**
Baseline	13.79M	87.63	97.98	96.13	79.62	97.97	93.34	68.80	99.60	92.89	84.46	99.15	95.10
Ours	28.74M	89.85	**99.43**	**97.46**	**80.23**	**99.51**	**94.70**	80.24	**99.70**	**94.99**	**87.03**	**99.35**	95.94

**Table 6:** Performance evaluation of Jaccard coefficient, Dice coefficient and Matthew correlation coefficient of different attention modules and CSFAG modules on the ISIC-2017 data set using baseline as the basic structure. Bold data indicates that the value is the maximum value in this indicator.

Method	Params	Nevus Cases			Melanoma Cases			SK Cases			Overall		
		DIC	JAC	MCC	DIC	JAC	MCC	DIC	JAC	MCC	DIC	JAC	MCC
SE Block+Baseline	26.62M	87.61	78.47	86.13	84.15	73.52	**78.79**	81.76	72.10	**80.91**	85.89	78.48	82.63
FPA+Baseline	32.08M	87.36	77.89	85.90	83.99	73.64	77.20	81.36	70.34	76.83	85.09	77.05	82.89
CBAM+Baseline	27.40M	88.55	79.45	86.74	**84.92**	72.91	77.90	82.47	71.17	78.98	85.16	77.15	82.38
Baseline	13.79M	87.14	78.16	85.56	83.31	72.13	77.11	78.19	62.80	73.85	84.74	76.65	81.78
Ours	28.74M	**90.65**	**82.90**	**89.19**	84.24	**73.68**	78.10	**82.14**	**72.78**	77.56	**86.97**	**78.85**	**83.93**

**Table 7:** The performance comparison of sensitivity, specificity and accuracy of the three basic architecture networks and their new network structure with the CSAG and DCCNet (ours) model on the ISIC-2017 data set. Bold data indicates that the value is the maximum value in this indicator.

Method	Params	Nevus Cases			Melanoma Cases			SK Cases			Overall		
		SEN	SPE	ACC	SEN	SPE	ACC	SEN	SPE	ACC	SEN	SPE	ACC
UNet	8.60M	81.80	97.53	93.83	70.31	98.08	89.62	57.84	98.34	89.07	74.53	97.59	92.32
UNet+CSFAG	23.15M	87.35	99.31	96.00	73.89	97.68	90.16	**82.98**	98.87	91.49	85.67	98.45	94.34
SegNet	28.08M	82.83	98.65	95.60	73.24	99.02	91.69	65.62	98.98	92.64	80.19	99.00	95.46
SegNet+CSFAG	42.78M	85.51	98.99	96.31	74.47	99.14	93.29	82.69	98.10	91.82	82.41	98.99	95.34
M-Net	10.92M	85.96	97.31	96.05	78.60	97.58	92.17	72.60	98.32	91.69	83.22	98.32	94.58
M-Net+CSFAG	24.89M	88.82	97.63	96.82	79.40	97.20	93.24	69.94	98.02	93.39	86.54	97.41	95.85
Ours	28.74M	**89.85**	**99.43**	**97.46**	**80.23**	**99.51**	**94.70**	80.24	**99.70**	**94.99**	**87.03**	**99.35**	**95.94**

**Table 8:** The performance comparison of Jaccard coefficient, Dice coefficient and Matthew correlation coefficient of the three basic architecture networks and their new network structure with the CSAG and DCCNet (ours) model on the ISIC-2017 data set. Bold data indicates that the value is the maximum value in this indicator.

Method	Params	Nevus Cases			Melanoma Cases			SK Cases			Overall		
		DIC	JAC	MCC	DIC	JAC	MCC	DIC	JAC	MCC	DIC	JAC	MCC
UNet	8.60M	78.71	64.89	75.12	73.16	57.68	66.73	64.39	47.49	58.55	74.32	59.13	69.64
UNet+CSFAG	23.15M	86.28	75.87	83.88	76.75	62.27	71.30	72.46	56.82	67.14	81.08	68.18	77.77
SegNet	28.08M	83.66	71.91	81.28	77.84	63.73	73.28	73.67	58.31	70.15	83.07	71.05	80.70
SegNet+CSFAG	42.78M	86.43	76.10	84.41	82.04	69.55	78.58	75.35	60.45	70.80	85.06	74.00	82.36
M-Net	10.92M	86.89	77.34	84.80	81.72	69.20	78.36	74.32	59.13	71.56	83.68	74.08	81.30
M-Net+CSFAG	24.89M	88.46	79.31	86.68	83.70	72.71	**79.83**	79.16	65.51	75.20	85.63	76.86	83.20
Ours	28.74M	**90.65**	**82.90**	**89.19**	**84.24**	**73.68**	78.10	**82.14**	**72.78**	**77.56**	**86.97**	**78.85**	**83.93**

**Table 9:** Performance evaluation of sensitivity, specificity, and accuracy performance of different attention modules and CSFAG modules on the ISIC-2017 data set using baseline as the basic structure. Bold data indicates that the value is the maximum value in this indicator.

Method	Nevus Cases			Melanoma Cases			SK Cases			Overall		
	SEN	SPE	ACC	SEN	SPE	ACC	SEN	SPE	ACC	SEN	SPE	ACC
U-Net [57]	76.76	97.26	92.89	58.71	96.81	84.98	43.81	97.64	84.83	67.15	97.24	90.14
FCN-AlexNet [58]	82.44	97.58	94.84	72.35	96.23	87.82	71.70	97.92	89.35	78.86	97.37	92.65
FCN-32s [58]	83.67	96.69	94.59	74.36	96.32	88.94	75.80	96.41	89.45	80.67	96.72	92.72
FCN-16s [58]	84.23	96.91	94.67	75.14	96.27	89.94	75.48	96.25	88.83	81.14	96.68	92.74
FCN-8s [58]	83.91	97.22	94.55	78.37	95.96	89.63	69.85	96.57	87.40	80.72	96.87	92.52
DeepLabV3+ [58]	88.54	97.21	95.67	77.71	96.37	89.65	74.59	98.55	90.06	84.34	97.25	93.66
Mask-RCNN [58]	87.25	96.38	95.32	78.63	95.63	89.31	82.41	94.88	90.85	84.84	96.01	93.48
SegNet [57]	85.19	96.30	93.93	73.78	94.26	87.90	70.58	92.50	87.29	80.05	95.37	91.76
FrCN [57]	88.95	97.44	95.62	78.91	96.04	90.78	82.37	94.08	91.29	85.40	96.69	94.03
Ensemble-A [58]	**92.08**	95.37	95.59	**84.62**	94.20	90.85	**87.49**	94.41	91.72	**89.93**	95.00	94.04
Ours	89.85	**99.43**	**97.46**	80.23	**99.51**	**94.70**	80.24	**99.70**	**94.99**	87.03	**99.35**	**95.94**

**Table 10:** Performance evaluation of Jaccard coefficient, Dice coefficient and Matthew correlation coefficient of different modules and CSFAG modules on the ISIC-2017 data set. Bold data indicates that the value is the maximum value in this indicator.

Method	Nevus Cases			Melanoma Cases			SK Cases			Overall		
	DIC	JAC	MCC	DIC	JAC	MCC	DIC	JAC	MCC	DIC	JAC	MCC
U-Net [57]	82.16	69.71	78.05	70.82	54.83	63.71	57.88	40.73	63.89	76.27	61.64	71.23
FCN-AlexNet [58]	85.61	77.01	82.91	75.94	64.32	70.35	75.09	63.76	71.51	82.15	72.55	78.75
FCN-32s [58]	85.08	76.39	82.29	78.39	67.23	72.70	76.18	64.78	72.10	82.44	72.86	78.89
FCN-16s [58]	85.60	77.39	82.92	79.22	68.41	73.26	75.23	64.11	71.42	82.80	73.65	79.31
FCN-8s [58]	84.33	76.07	81.73	80.08	69.58	74.39	68.01	56.54	65.14	81.06	71.87	77.81
DeepLabV3+ [58]	88.29	81.09	85.90	80.86	71.30	76.01	77.05	67.55	74.62	85.16	77.15	82.28
Mask-RCNN [58]	88.83	80.91	85.38	80.28	70.69	74.95	80.48	70.74	76.31	85.58	77.39	81.99
SegNet [57]	85.69	74.97	81.84	79.11	65.45	71.03	72.54	56.91	64.32	82.09	69.63	76.79
FrCN [57]	89.68	81.28	86.90	84.02	72.44	77.90	81.83	69.25	76.11	87.08	77.11	83.22
Ensemble-A [58]	89.28	82.11	86.33	83.54	**74.53**	78.08	**82.53**	**73.45**	**78.61**	**87.14**	**79.34**	83.57
Ours	**90.65**	**82.90**	**89.19**	**84.24**	73.68	**78.10**	82.14	72.78	77.56	86.97	78.85	**83.93**

**Table 11:** Performance evaluation of accuracy, Dice coefficient, Jaccard coefficient, sensitivity, specificity, and performance of different modules and CSFAG modules on the ISIC-2017 data set. Bold data indicates that the value is the maximum value in this indicator.

Model	Year	ACC	DIC	JAC	SEN	SPE
CDNN [59]	2017	0.934	0.849	0.765	0.825	0.975
SLSDeep [60]	2018	0.936	0.878	0.782	0.816	0.983
Jahanifar M et al. [61]	2018	0.930	0.839	0.749	0.810	0.981
Bi L et al. [62]	2019	0.862	0.857	0.777	**0.967**	0.941
Att-DenseUnet [63]	2019	0.9329	**0.8786**	**0.8035**	0.8734	0.9314
DAGAN [64]	2020	0.935	0.859	0.771	0.835	0.976
CSARM-CNN [65]	2020	0.958	0.846	0.733	0.802	**0.994**
DSC [66]	2020	0.938	0.862	0.783	0.870	0.964
Ours		**0.959**	0.869	0.788	0.870	0.993

**Table 12:** Performance evaluation of sensitivity, specificity, and accuracy performance of different attention modules and CSFAG modules on the ISIC-2017 data set using baseline as the basic structure. Bold data indicates that the value is the maximum value in this indicator.

Method	Nevus Cases			Melanoma Cases			Overall		
	SEN	SPE	ACC	SEN	SPE	ACC	SEN	SPE	ACC
FCN [47]	**95.35**	94.09	94.44	90.30	94.02	92.82	90.30	94.02	92.82
SegNet [47]	91.57	96.57	95.19	75.50	96.83	86.04	86.53	96.61	93.36
U-Net [47]	86.68	97.63	94.60	70.58	**98.47**	84.36	81.63	**97.76**	92.55
FrCn [47]	94.48	95.46	95.20	**91.57**	96.55	**94.64**	**93.72**	95.65	95.08
Ours	93.30	**98.19**	**97.35**	85.34	95.72	91.00	89.75	97.73	**95.90**

**Table 13:** Performance evaluation of Jaccard coefficient, Dice coefficient and Matthew correlation coefficient of different attention modules and CSFAG modules on the ISIC-2017 data set using baseline as the basic structure. Bold data indicates that the value is the maximum value in this indicator.

Method	Nevus Cases			Melanoma Cases			Overall		
	DIC	JAC	MCC	DIC	JAC	MCC	DIC	JAC	MCC
FCN [47]	90.46	82.59	86.78	89.03	80.22	83.71	89.03	80.22	83.71
SegNet [47]	91.32	84.03	87.99	84.55	73.23	73.89	89.36	80.77	84.64
U-Net [47]	89.88	81.63	86.32	82.04	69.55	71.73	87.61	77.95	82.78
FrCn [47]	91.38	84.13	88.15	**92.92**	**86.77**	**88.62**	**91.77**	**84.79**	**88.30**
Ours	**92.37**	**85.83**	**90.78**	89.61	83.87	85.02	90.97	83.44	88.34

## Data Availability

We used two public dermoscopy datasets to evaluate the proposed segmentation network, namely the ISIC-2017 challenge dataset [52] and the PH2 dataset [53]. The ISIC-2017 challenge data set is provided by the International Skin Imaging Collaboration (ISIC). The PH2 data set was collected by the Pedro Hispano Department of Dermatology in the hospital and the Técnico Lisboa University of Porto research team in Matosinhos, Portugal. The url of ISIC2017 is https://challenge.isic-archive.com/landing/2017, accessed on 16 May 2021.

## Abbreviations

The following abbreviations are used in this manuscript:

CSAG and DCCNet	Channel Spatial Attention-guided Network with Densely Connected Convolutional
CSFAG	Channel Spatial Fast Attention-guided Filter
FCN	Full Convolutional Network
DCNN	Deep Convolutional Neural Network
SEN	Sensitivity
SPE	Specificity
ACC	Accuracy
JAC	Jaccard
DIC	Dice Coefficient
MCC	Matthew Correlation Coefficient
